# The Feasibility and Acceptability of Training Dietitians to Hand Feed People Living With Dementia

**DOI:** 10.1093/geroni/igaa057.1444

**Published:** 2020-12-16

**Authors:** Christine Ferguson, Joy Douglas, Beth Nolan

**Affiliations:** 1 The University of Alabama, Tuscaloosa, Alabama, United States; 2 Positive Approach to Care, Ada, Michigan, United States

## Abstract

The purpose of this study was to evaluate the feasibility and acceptability of a training program using the interactive Positive Approach to Care□ dementia care curriculum to Registered Dietitians (RDs). To recruit RDs, the Alabama Dietetic Association emailed its member twice about the training opportunity, and a maximum of 25 potential participants could register online. Of those who registered, 80% (20/25) attended the training, and all attendees agreed to the informed consent. The total cost for the two training sessions, including travel, supplies, and labor hours, was approximately $800. The primary challenge for the facilitator was accurately following the script due to time constraints. Two weeks after completing the training, participants answered open ended questions, and many shared how they enjoyed the hands-on activities and watching videos of people living with dementia being fed using the Hand-Under-Hand□ technique. Some participants would have preferred a longer workshop at a location that was more convenient for them. All participants shared how this training may impact their professional practice, such as how it changed the way they approach and communicate with people living with dementia. Since receiving the training, many shared how they have either already started or are interested in sharing the information with interdisciplinary healthcare professionals and/or caregivers. Overall, the training session is considered a feasible, acceptable, and low-cost approach to training RDs on providing hands-on care to people living with dementia. Lengthening the training may improve the replicability of the script in addition to providing opportunities for more hands-on activities.

